# Supplementary figures and images for: Information theoretic measures of neural and behavioural coupling predict representational drift
Source: PLoS Comput Biol. 2026 Feb 17;22(2):e1013130. doi: 10.1371/journal.pcbi.1013130 (PMC12952586; doi:10.1371/journal.pcbi.1013130)

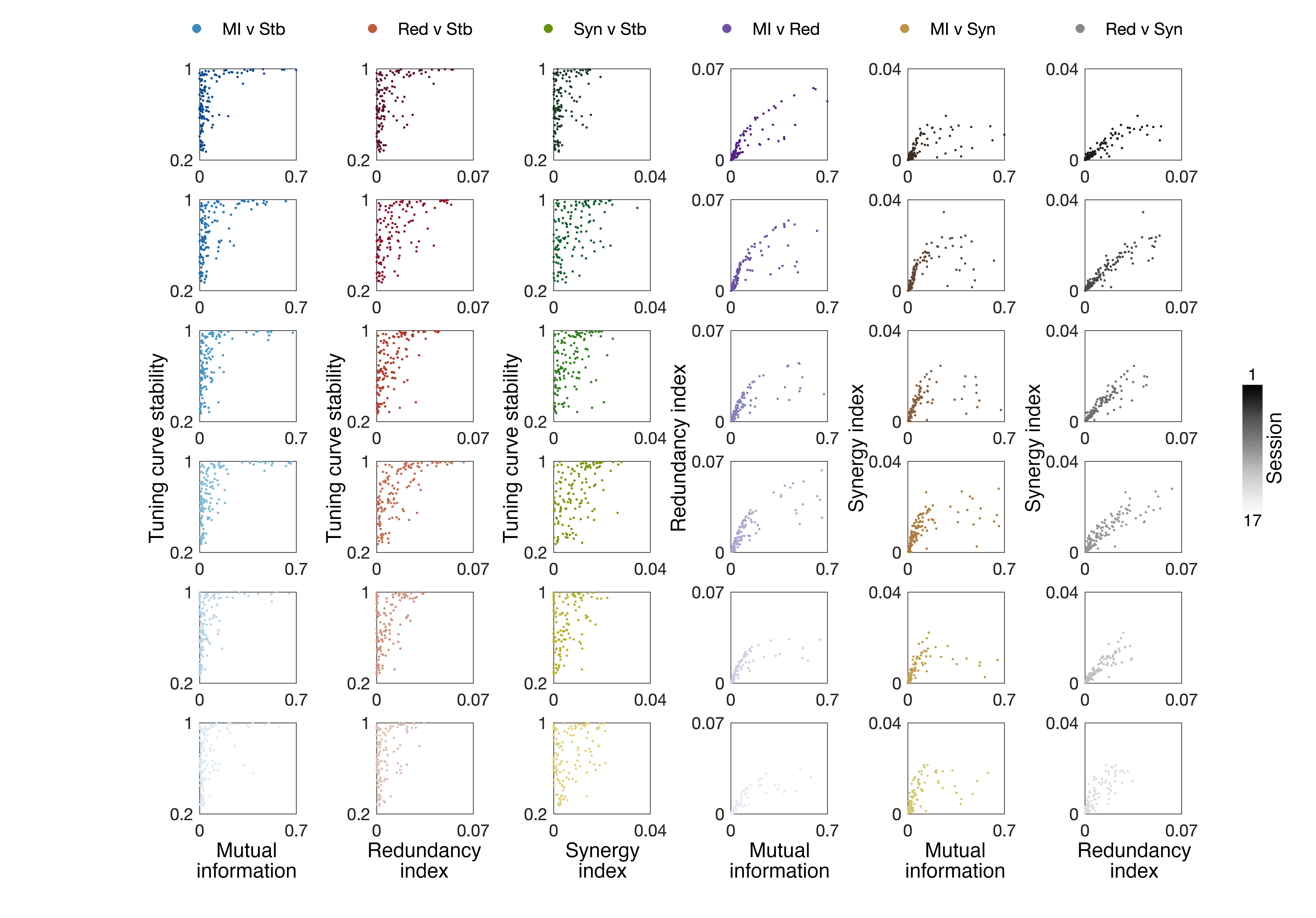

Supplement: S1 Fig — Example pairwise plots of considered variables for one mouse (mouse 4, PPC, external variable: position, left-turn trials) on different sessions. Each data point represents one neuron (n = 194 neurons). Rows show a selection of six sessions (sessions 1, 4, 7, 10, 13, and 16) out of a total of 17 sessions. Columns show a given pairwise plotting. The four plotted variables are the tuning stability, mutual information, redundancy index, and synergy index. In the regressions performed in this study, these are respectively the response variable and the three predictors. (TIFF) [file pcbi.1013130.s001.tif]

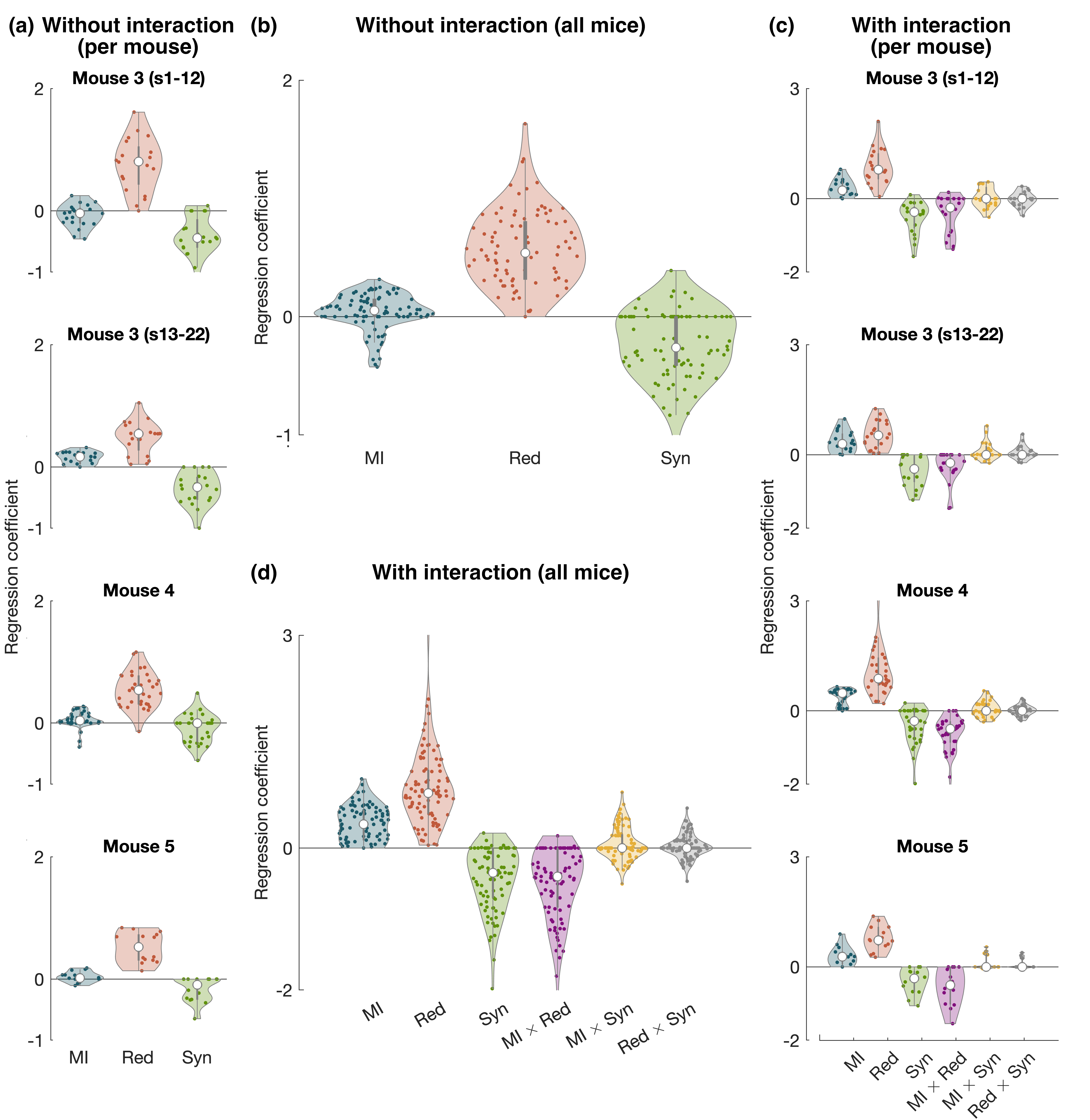

Supplement: S2 Fig — Comparison of regression coefficients obtained by elastic net (a,b) without and (c,d) with interaction terms. In the regressions, the response variable was the tuning stability, and the three predictors were mutual information (MI), redundancy index (Red), and synergy index (Syn), with maze position as the target external variable. Each data point plotted here represents regression coefficients for one mouse on a single session. (a,c) Regression coefficients plotted per neural population: mouse 3, 194 cells, 10 sessions (sessions 1, 2, 4, 6–12); mouse 3, 177 cells, 10 sessions (sessions 13–22); mouse 4, 130 cells, 17 sessions (sessions 1–17); mouse 5, 130 cells, 7 sessions (sessions 7–13). (b,d) Regression coefficients across all mice. Elastic net was performed using MATLAB’s lasso function. (TIFF) [file pcbi.1013130.s002.tif]

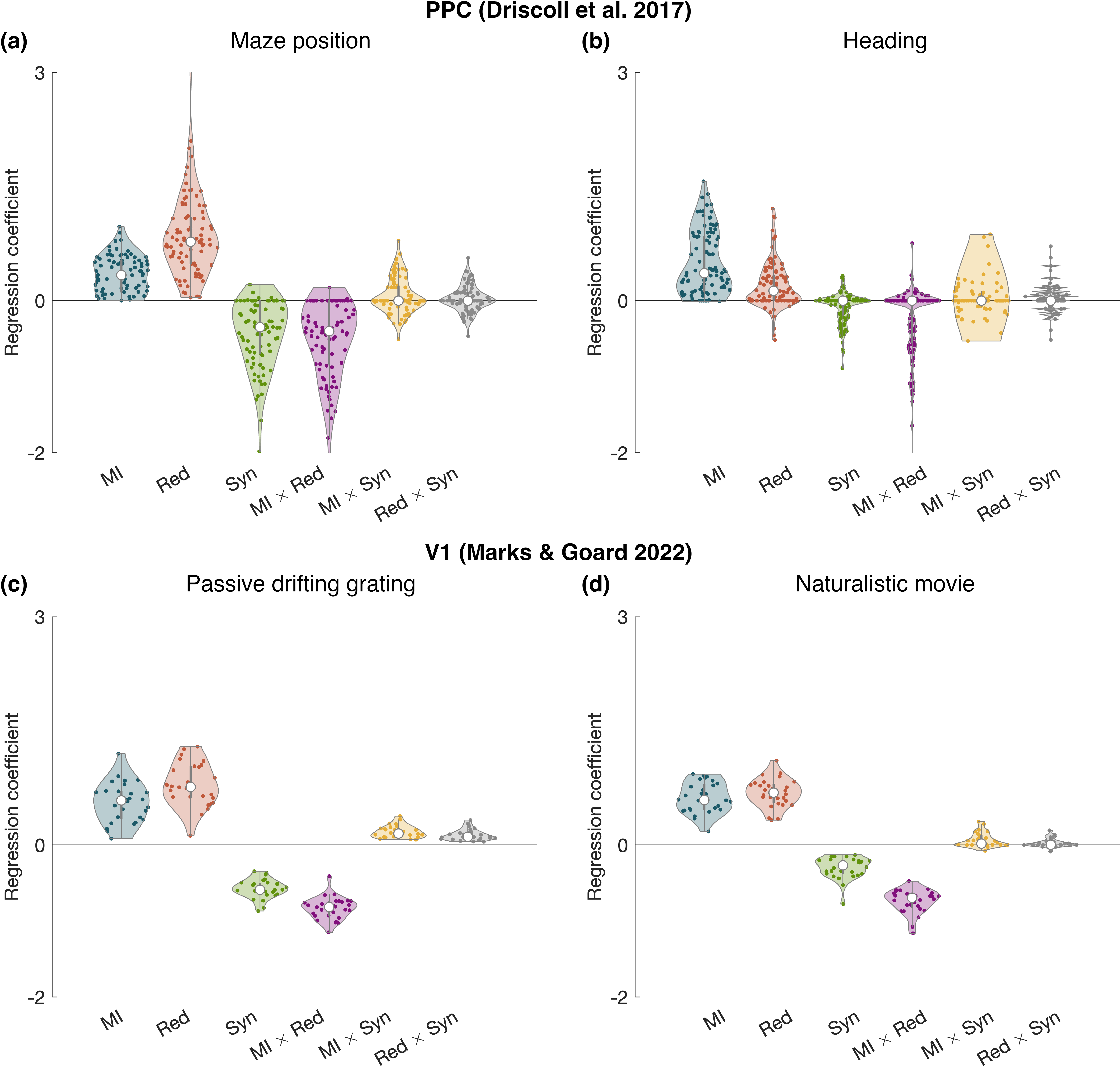

Supplement: S3 Fig — Regression results are shown for each considered external variable: (a) position, (b) heading, (c) gratings, and (d) movies. (TIFF) [file pcbi.1013130.s003.tif]

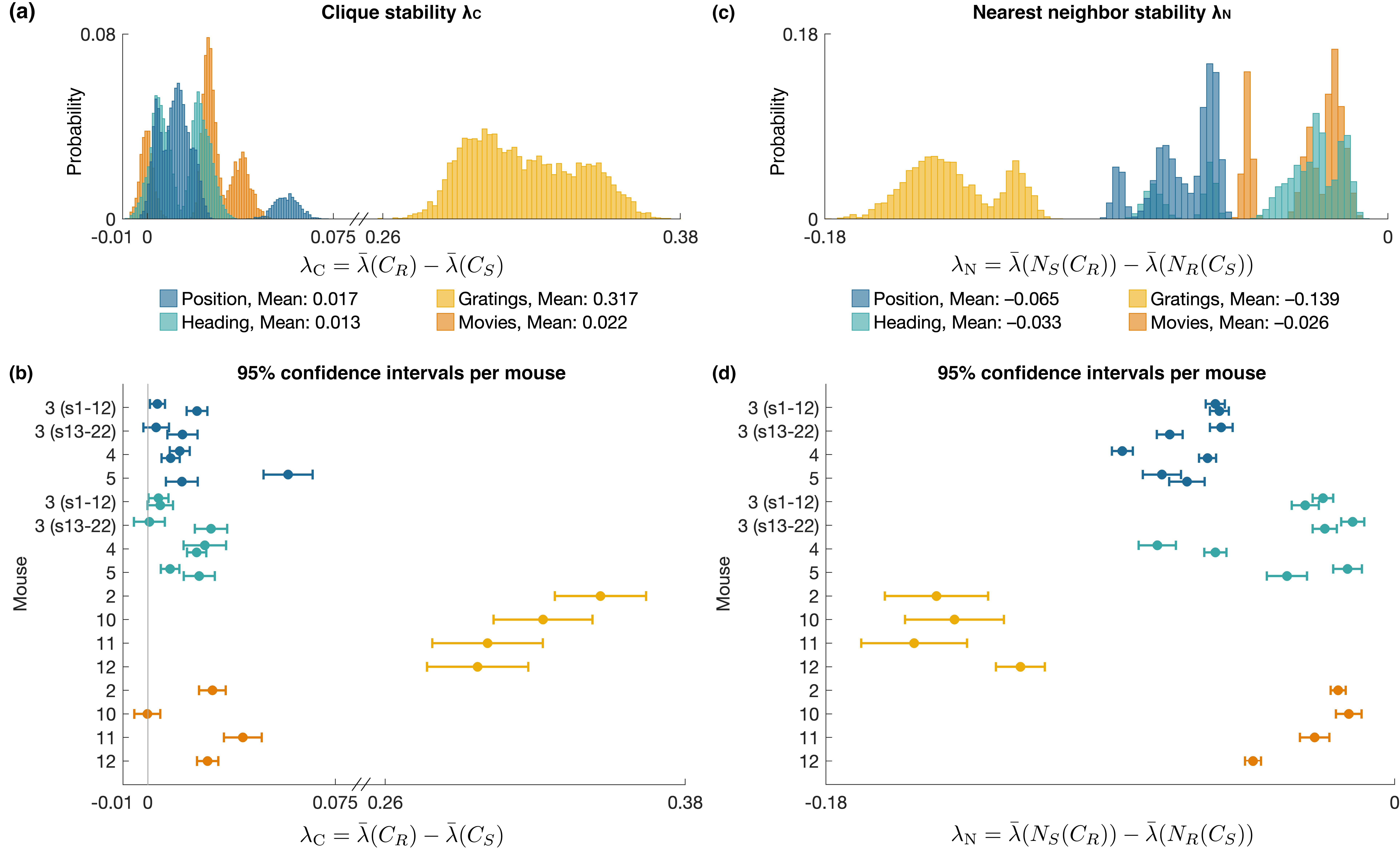

Supplement: S4 Fig — Histograms and 95% confidence intervals of means from 1,000 dataset replicants generated by bootstrap resampling with replacement. (a) Difference in mean stability of size-matched redundant and synergistic cliques, λC=λ¯(CR) − λ¯(CS). Histograms are pooled over mice. (b) Mean and 95% confidence intervals per mouse. For U the position or heading, left- and right-turn trials are plotted separately. (c,d) Same as (a,b) for difference in mean stability of clique nearest neighbors in opposing graph, λC=λ¯(CR) − λ¯(CS). (TIFF) [file pcbi.1013130.s004.tif]

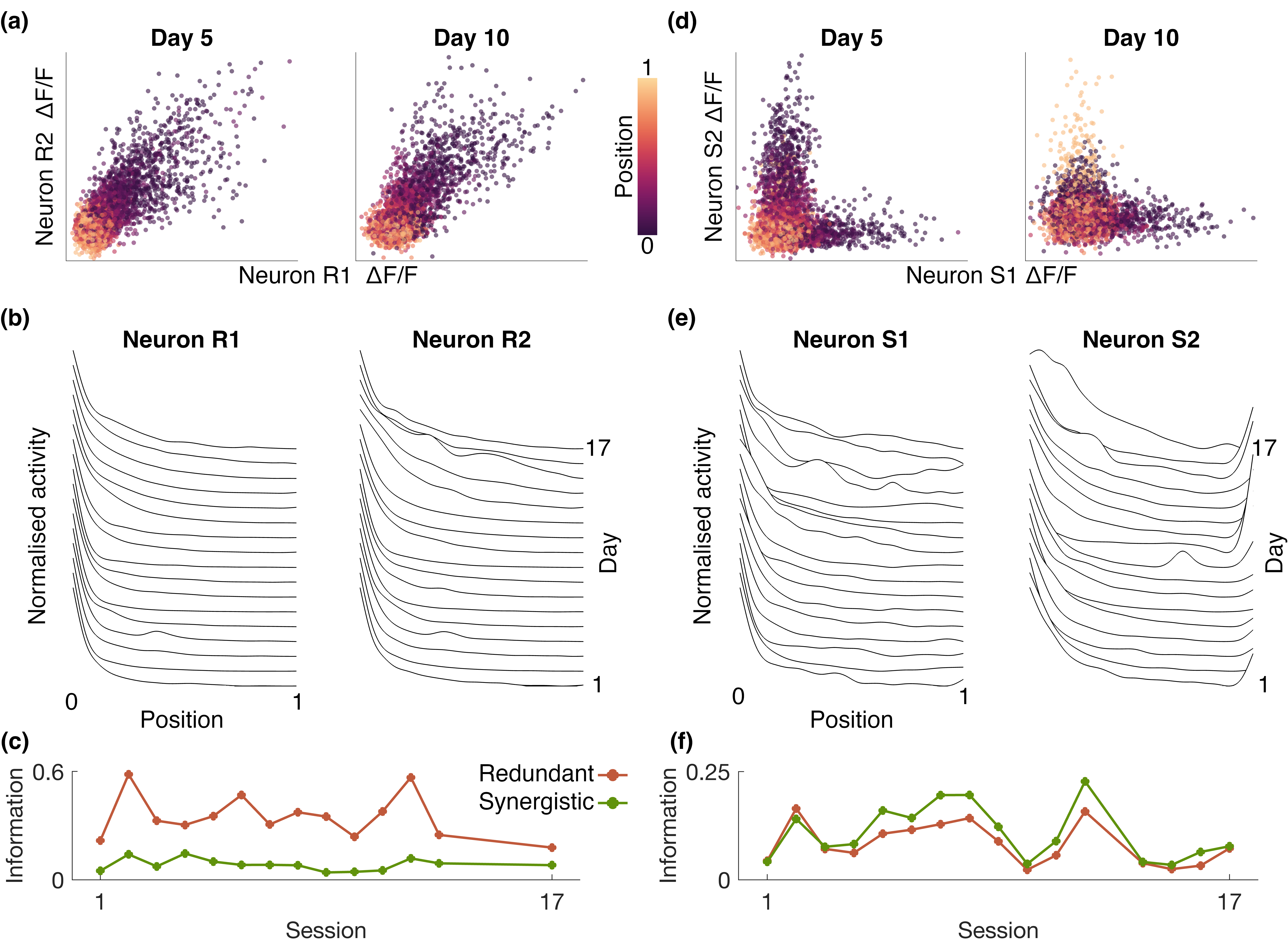

Supplement: S5 Fig — These pairs correspond to those shown in Fig 1 (PPC dataset, external variable: position, mouse 4). (a) Neural activity of a highly redundant neuron pair, Neurons R1 and R2, on two different sessions. ΔF/F samples are colored according to the normalised position in the maze. (b) Tuning curves of the two neurons in (a), with sessions stacked vertically. (c) Redundant and synergistic information for the pair shown in (a) plotted over sessions. (d)–(f) Same as (a)–(c) for a pair with high synergistic information, Neurons S1 and S2. (TIFF) [file pcbi.1013130.s006.tif]

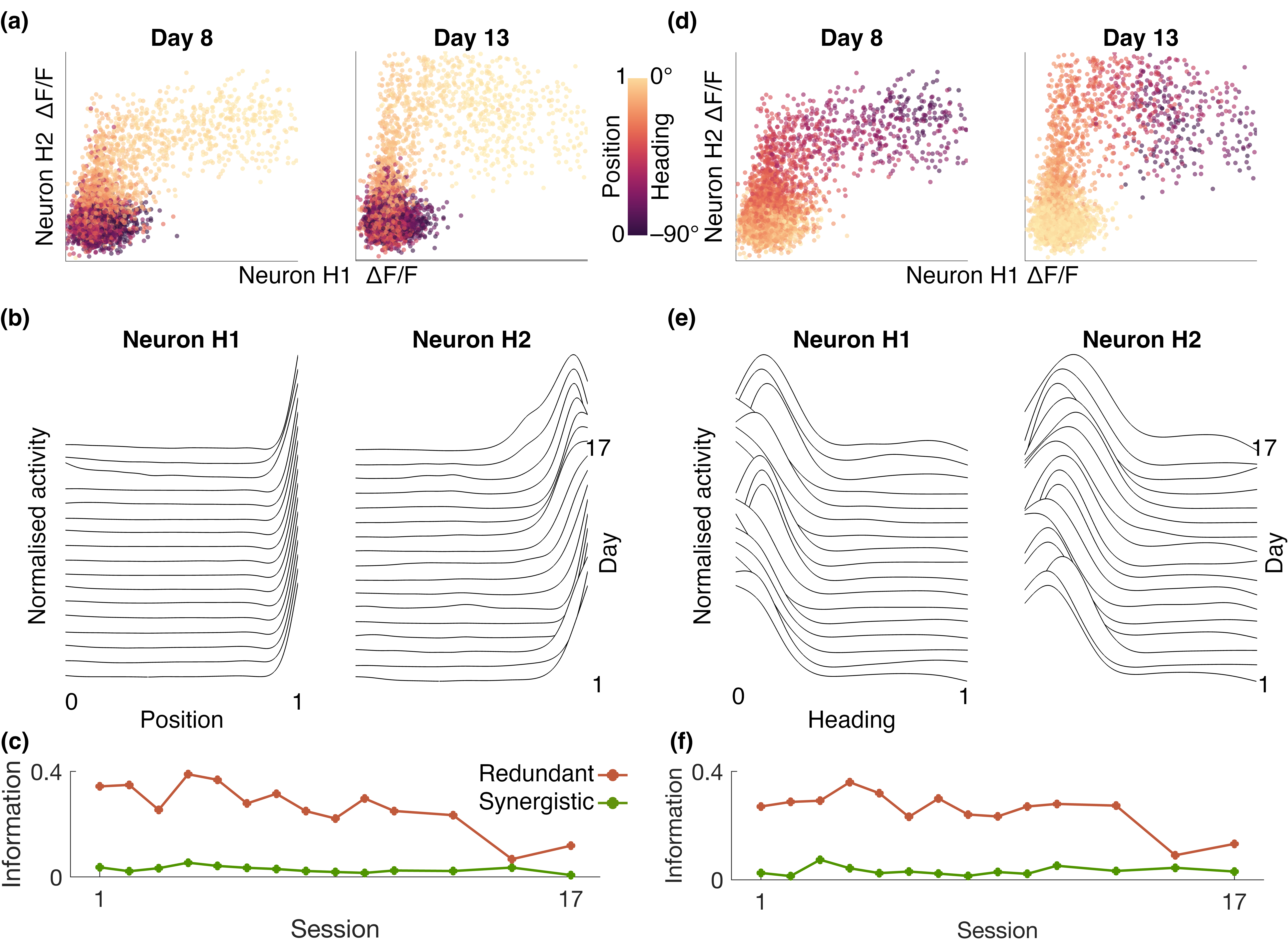

Supplement: S6 Fig — Same as S5a-c Fig for a neuron pair with high redundancy, Neurons H1 and H2. (d)–(f) Same as (a)–(c) but with heading, instead of position, as the external variable. (TIFF) [file pcbi.1013130.s007.tif]
