# Supplementary material for: Information theoretic measures of neural and behavioural coupling predict representational drift
Source: PLoS Comput Biol. 2026 Feb 17;22(2):e1013130. doi: 10.1371/journal.pcbi.1013130 (PMC12952586; doi:10.1371/journal.pcbi.1013130)
Supplement: S1 Table — Parameters of graphs used to compare clique {CR,CS} stability and clique nearest neighbour {NS(CR),NR(CS)} stability in S4 Fig. See Methods for details. Clique size is given as a range of percentages of the total number of cells. The number of R–S pairs gives the range of the per-session number of paired unweighted R and S graphs with equal clique sizes. (PDF) [file pcbi.1013130.s005.pdf]

S1 Table: Graph analysis parameters for Figs 3 and S4. Parameters of graphs used to compare clique $\{C_R, C_S\}$ stability and clique nearest neighbour $\{N_S(C_R), N_R(C_S)\}$ stability in Figs 3 and S4. See Methods for details. Clique size is given as a range of percentages of the total number of cells. The number of R–S pairs gives the range of the per-session number of paired unweighted R and S graphs with equal clique sizes.

Brain region	Edge thresholds	Task variable	Mouse, sessions	Clique size [% pop.]	Number of R–S pairs
PPC	0.25–25%	Position	m3, s1–12	2–38%	29–45
			m3, s13–22	3–38%	25–44
			m4, s1–17	3–40%	21–38
			m5, s7–13	3–39%	20–37
		Heading	m3, s1–12	2–37%	21–47
			m3, s13–22	2–34%	13–39
			m4, s1–17	3–41%	15–37
			m5, s7–13	3–28%	11–27
V1	0.25–15%	Gratings	m1, s1–7	1–20%	16–19
			m10, s1–7	1–24%	12–19
			m11, s1–7	1–22%	11–17
			m12, s1–7	1–16%	15–26
		Movies	m1, s1–7	1–19%	18–27
			m10, s1–7	1–19%	13–18
			m11, s1–7	2–23%	12–19
			m12, s1–7	1–13%	15–24
