# Supplementary material for: Information theoretic measures of neural and behavioural coupling predict representational drift
Source: PLoS Comput Biol. 2026 Feb 17;22(2):e1013130. doi: 10.1371/journal.pcbi.1013130 (PMC12952586; doi:10.1371/journal.pcbi.1013130)
Supplement: S1 Text — (PDF) [file pcbi.1013130.s008.pdf]

S1 Text: Toy example of synergy

In this appendix, we present a toy example of how synergy with respect to task variable U can arise from a pair of neurons having opposite co-tuning to an unobserved task variable V.

In this toy example, we consider two neurons with similar tuning to U and opposite tuning to V. To relate this toy example to one of the datasets we analysed (Driscoll et al. 2017), let us say $V = \{0, 1\}$ indicates that a trial is a left- or right-turn trial, respectively, and U is the position, with two position bins for the sake of simplicity. Let us consider the case where neurons X and Y are both tuned to position bin $U = 0$, but neuron X is active in only right-turn trials ($V = 1$) and neuron Y in only left-turn trials ($V = 0$). This gives the following probability tables for U and V taken individually:

U	X	Y	$p(X, Y\|U)$
0	0	0	1/2
0	0	1	1/4
0	1	0	1/4
0	1	1	1/2
1	0	0	1
1	0	1	0
1	1	0	0
1	1	1	0

V	X	Y	$p(X, Y\|V)$
0	0	0	3/4
0	0	1	1/4
0	1	0	0
0	1	1	0
1	0	0	3/4
1	0	1	0
1	1	0	1/4
1	1	1	0

Preserving the active probabilities and assuming $U = \{0, 1\}$ and $V = \{0, 1\}$ occur independently and with equal probabilities, we can construct a joint probability table:

$V = 0$				$V = 1$			
U	X	Y	$p(X, Y\|U)$	U	X	Y	$p(X, Y\|U)$
0	0	0	1/2	0	0	0	1/2
0	0	1	1/4	0	0	1	0
0	1	0	0	0	1	0	1/4
0	1	1	0	0	1	1	0
1	0	0	1	1	0	0	1
1	0	1	0	1	0	1	0
1	1	0	0	1	1	0	0
1	1	1	0	1	1	1	0

First, an intuition-based explanation. The toy example here resembles a discretised version of the activity shown in Fig. 1(h) and (i): when the animal is in position $u = 1$ (yellow), the neurons are inactive ($\{X, Y\} = \{0, 0\}$), and in position $u = 0$ (purple), one or the other is active ($\{X, Y\} = \{1, 0\}$ or $\{0, 1\}$). This has the effect of better separating the regions of XY space where $U = 0$ and $U = 1$ than if the activity of X and Y were uncorrelated, as illustrated in Fig. 1(i).

For a more mathematically rigorous explanation, we can calculate the synergistic from Eq. (9) in the Methods, using the formulation by Bertschinger et al. (2014):

$$\tilde{I}_{\text{syn}}(U : X\,;\,Y) = I(U : X, Y) - \min_{Q \in \Delta_P} I_Q(U : X, Y). \tag{1}$$

We can calculate the joint mutual information from the probability table for U as $I(U; X, Y) = 3/2 - 3/4 \log_2 3 = 0.31$ bit. The second term is the minimal joint mutual information among the set of probability distributions Δ_P that maintains the tuning curve relationships between each neuron and U (i.e., the marginal distributions $p(U, X)$ and $p(U, Y)$ are the same). This distribution would be the case where the two neurons are always active together in the position bin to which they are tuned:

U	X	Y	$p_Q(X,Y\|U)$
0	0	0	$^3/_8$
0	0	1	0
0	1	0	0
0	1	1	$^1/_8$
1	0	0	1
1	0	1	0
1	1	0	0
1	1	1	0

The joint mutual information for this probability distribution is $I_Q(U;X,Y) = 2 + {}^3/_8 \log_2 3 - {}^7/_8 \log_2 7 = 0.14$ bit. The synergistic information is then the difference between these two, which gives us $\tilde{I}_{\text{syn}} = 0.17$ bit. Note that the same results come from assuming the two neurons mutually inhibit one another, as the probability table for U would be the same.
