# Supplementary material for: Information theoretic measures of neural and behavioural coupling predict representational drift
Source: PLoS Comput Biol. 2026 Feb 17;22(2):e1013130. doi: 10.1371/journal.pcbi.1013130 (PMC12952586; doi:10.1371/journal.pcbi.1013130)
Supplement: Attachment — Submitted filename: PCOMPBIOL-D-25-00928_review.pdf [file pcbi.1013130.s009.pdf]

Review of the manuscript PCOMPBIOL-D-25-00928, “Information theoretic measures of neural and behavioural coupling predict representational drift” by Heiney et al.

Neural selectivity to behavioral variables changes across day even when the task remains stable, a phenomenon called representation drift. In this manuscript, Heiney et al. analyze how the coding properties of a neuron, and its relationship with other neurons, correlate with its changes in selectivity across time. Using modern information theory tools (Partial Information Decomposition), the authors suggest that neurons with larger stimulus information shared with other neurons (measured by the redundancy), tends also to be exhibit a more stable tuning to the same stimulus across days, suggesting an interesting relationship between information theoretic measures and principles of representation drift.

I find it an interesting study, that addresses an important question with solid analytical tools, and I think it will be of interest to readership of Plos Computational Biology.
However, the scope of the contribution is somewhat limited in its current form, and I believe that the paper would be benefit from some more comprehensive analyses.

Main comments

The main result of the paper is that the stability of a neuron i, quantified by its change of tuning across days, correlates positively with the redundancy index of a neuron, quantified by the pairwise redundancy for each pair ij, averaged across j. I find this to be an interesting result, but I find unclear some related discussions.

The claim in line 179, ’’Interestingly, the coefficient for the synergy index tended to be negative. This means that among neurons with a given redundancy index, a neuron with a higher synergy index would tend to exhibit lower tuning stability.“, is not fully supported by the presented analyses. I believe that, to support the claim about the conditional effect of synergy index given the redundancy, the authors should fit a statistical model with an explicit interaction term Syn*Red.

In addition, the paragraph in the Discussion between lines 221-224 is weakly supported by the presented analyses. A more compelling analysis might involve examining the redundancy and synergy graphs in Fig. 2 d, to eventually detect cliques and structures. More generally, the analysis is currently restricted to a single neuron (or “node”), measure i.e., the average of pairwise redundancy. A network approach, somehow already sketched in Fig 2d, would help to scale the results to pairwise relationships and toward a population-level interpretation, in line with recent studies such as Nigam et al., *Neuron* 2019, and Safaai et al., *bioRxiv* 2023.

Minor comments

As far as I understand (and this could be clarified more explicitly in the Methods), the reported results are obtained by averaging information and drift measures across all pairs of

days. It would be valuable to assess whether the reported effects also hold at the level of individual session pairs. For example, does the redundancy index of a neuron on day *n* predict its tuning stability on day *n+1*? Such an analysis would enhance the predictive strength of the findings and would nicely complement the study by Eppler et al.,2024 “Representational drift reflects ongoing balancing of stochastic changes by Hebbian learning”, which uses signal and noise correlations to predict neuronal stability.

In limited sampling scenarios, PID measures are biased and the bias affect the components in a different way, with the synergy being more affected (Venkatesh et al., Neurips 2023, Lorenz et al., Plos CompBio 2025); did the authors control for this/took this into account (even with some shuffling method)?

I have a remark on the notation: typically uppercase letters are used to random variables, while lowercase letters denote their realization; is there a reason why they are reversed here?

The github link is broken and I cannot find the code; maybe they forgot to make the repo public?
