# Supplementary material for: Information theoretic measures of neural and behavioural coupling predict representational drift
Source: PLoS Comput Biol. 2026 Feb 17;22(2):e1013130. doi: 10.1371/journal.pcbi.1013130 (PMC12952586; doi:10.1371/journal.pcbi.1013130)
Supplement: Attachment — Submitted filename: Reviews.pdf [file pcbi.1013130.s010.pdf]

Overview

This manuscript addresses a key open question regarding the link between population interactions and representational drift phenomena. The long-term goal of this research is to predict which couplings (among and between neurons and behaviors) are likely to remain stable, and how they relate to changes in population coding and drift.

This is a methodologically rigorous manuscript that tackles an open issue in systems and network neuroscience. However, I have several major concerns that I would like the authors to address. These concerns primarily pertain to the presentation of the working hypotheses, the description of the results, and the conclusion. No additional major analyses are required.

Major Concerns

1. Working Hypothesis

I have concerns about the working hypotheses proposed in the introduction. As I understand it, the authors suggest that neurons conveying redundant information about core task or stimulus features are more constrained and stable over time, and thus less susceptible to representational drift. However, I would argue that neurons conveying highly redundant information could still exhibit strong representational drift, provided that their activity changes in a similar way—for instance, due to modulation over time of shared inputs. Conversely, one could argue that neurons conveying strong synergistic information about task variables should show greater stability over time. Given their heterogeneous coding, even slight representational shifts could cause the synergistic information to diminish.

In other words, the current working hypothesis does not appear to be strongly supported by empirical evidence or theoretical rationale. I suggest revising the final paragraph of the introduction to acknowledge and account for these alternative hypotheses. The question addressed in the paper is important, but I find the working hypothesis less convincing in its current form.

2. Statistical Analysis of Information Measures

The manuscript focuses on highly redundant and synergistic neuron pairs. However, no statistical analysis was performed to determine the significance of the main effects or to validate the identification of these neuron pairs. A common approach to assess significance in PID measures is to use surrogate data by shuffling the target variable (e.g., task-related variables). Additionally, any analysis should account for multiple comparisons, for example by applying FDR or Bonferroni corrections. The authors should consider performing such analyses or using quantitative methods to support their claims about highly redundant/synergistic neuron pairs.

3. Figure 1

Panel (a) contains a large amount of information, and the corresponding description in the results section is neither linear nor clear. The text reads more like a figure legend than a results narrative. I recommend rewriting this section to clearly describe the main results and streamline the narrative. Also, consider removing non-essential information (e.g., panel c). The definition of highly redundant pairs appears to overlap with standard linear correlation (see Fig. 1d). However, this might not always be the case. For example, panels (e) and (i) show correlation and anti-correlation, both of which could correspond to redundant information.

Additionally, panel (b) introduces the Redundancy Index before it is defined in the main text. Overall, I suggest revisiting Figure 1 entirely—selecting the most relevant results and providing a clearer, more structured explanation in the results section.

4. Statistical Relevance of Predictors of Drift

The description of the regularized regression procedure is insufficient. The manuscript should provide a more thorough and accessible explanation of the method and include references. For example, it should be clarified that the elastic net minimizes a specific loss function, combining L1 and L2 penalties. It is particularly useful for constructing linear models when predictors are numerous and potentially correlated.

5. Synergistic Information and Representational Drift

The negative correlation between synergistic information and representational drift is intriguing and could represent an important result. However, the manuscript emphasizes redundancy encoding results, and this potentially significant finding receives little attention. I suggest the authors highlight and elaborate on this finding more thoroughly.

6. Conclusion

The conclusion is currently very brief and does not reflect the full scope of the main findings. A more comprehensive conclusion summarizing key points—including those related to synergistic coding and the statistical predictors of drift—would strengthen the manuscript.

Minor Issues

- Important references on Partial Information Decomposition (PID) are missing.
- The code availability section lacks the link: https://github.com/kris-heiney/PID-Drift/

Conclusion

This is a methodologically rigorous manuscript that addresses a key open issue in systems and network neuroscience. However, addressing the concerns outlined above would greatly strengthen the clarity, robustness, and overall impact of the study.
