# Supplementary material for: Information theoretic measures of neural and behavioural coupling predict representational drift
Source: PLoS Comput Biol. 2026 Feb 17;22(2):e1013130. doi: 10.1371/journal.pcbi.1013130 (PMC12952586; doi:10.1371/journal.pcbi.1013130)
Supplement: Attachment — Submitted filename: Response to reviewers.docx [file pcbi.1013130.s011.docx]

**Response to reviewers**

**Journal**: PLOS Computational Biology

**Manuscript**: Information theoretic measures of neural and behavioural coupling predict representational drift

**Authors**: Kristine Heiney, Mónika Józsa, Michael E. Rule, Henning Sprekeler, Stefano Nichele, Timothy O’Leary

We would like to thank the editors for taking the time to consider our manuscript, and the reviewers for their insightful and helpful comments. In following with the reviewers’ comments, we have performed some additional analyses and made changes to the manuscript, and we believe that the manuscript has improved as a result.

Please find below our response to each of the reviewers’ comments.

**Reviewer 1**

***Major comments***

The claim in line 179, ‘’Interestingly, the coefficient for the synergy index tended to be negative. This means that among neurons with a given redundancy index, a neuron with a higher synergy index would tend to exhibit lower tuning stability.”, is not fully supported by the presented analyses. I believe that, to support the claim about the conditional effect of synergy index given the redundancy, the authors should fit a statistical model with an explicit interaction term Syn*Red.

We have added an additional stepwise regression analysis to support our elastic net analysis and better evaluate the interactions among the predictor variables. The methods are described in the corresponding methods section (‘Statistical relevance of predictors of drift’), and the results are shown in Supplementary Figures S2 and S3.

In our stepwise regression analysis, we initialised our regression model with six terms: three linear terms for the three predictors and three interaction terms. We then removed the least significant terms by backward elimination. The resultant coefficients were qualitatively consistent with the elastic net results, with redundancy index showing higher positive coefficients than mutual information (except in the heading case) and synergy index tending to exhibit negative coefficients.

The interaction results revealed more complex relationships among the variables than we anticipated. In the PPC dataset with position as the external variable, the initial statement we made held: the Red*Syn term tended to have a negative coefficient. However, in the V1 dataset, this coefficient was positive or zero. We have amended our claims in the results to reflect that our original statement only holds for the PPC dataset with U=position.

While comparing the stepwise and elastic net regression results, we also realised that we mistakenly included one of the mice in PPC dataset that did not meet our inclusion criteria (mouse 1, fewer than 100 neurons). Figure 2(b) has been updated with this mouse excluded; this exclusion does not substantially change our results.

In addition, the paragraph in the Discussion between lines 221-224 is weakly supported by the presented analyses. A more compelling analysis might involve examining the redundancy and synergy graphs in Fig. 2 d, to eventually detect cliques and structures. More generally, the analysis is currently restricted to a single neuron (or “node”), measure i.e., the average of pairwise redundancy. A network approach, somehow already sketched in Fig 2d, would help to scale the results to pairwise relationships and toward a population- level interpretation, in line with recent studies such as Nigam et al., Neuron 2019, and Safaai et al., bioRxiv 2023.

We have amended this statement as, ‘Our results demonstrate that the most stable cells in a population tend to participate in redundantly connected cliques that in turn synergistically connect to the rest of the population’, and we have included additional graph analysis to support this statement. The details are provided in a new methods section (‘Clique detection and graph analysis’).

In short, we detected cliques (fully connected subgraphs, illustrated in Fig S4(a)) in the redundant and synergistic graphs and compared their average stability. We then compared the average stability of the nearest neighbours of the cliques in their opposing graphs (synergistic neighbours of redundant cliques and vice versa, schematically illustrated in Fig S4(c)). We found that neurons forming redundant cliques tend to have higher stability than those forming synergistic cliques (Fig S4(b), Table S1), and that the neighbours of redundant cliques in the synergistic graph tend to have lower stability than the neighbours of synergistic cliques in the redundant graph (Fig S4(d)).

***Minor comments***

As far as I understand (and this could be clarified more explicitly in the Methods), the reported results are obtained by averaging information and drift measures across all pairs of days. It would be valuable to assess whether the reported effects also hold at the level of individual session pairs. For example, does the redundancy index of a neuron on day n predict its tuning stability on day n+1? Such an analysis would enhance the predictive strength of the findings and would nicely complement the study by Eppler et al.,2024 “Representational drift reflects ongoing balancing of stochastic changes by Hebbian learning”, which uses signal and noise correlations to predict neuronal stability.

The reported regression results are obtained by performing a regression for each individual session. The drift measure is indeed averaged over all session pairs, but the information measures are taken session-wise to avoid biasing the results—that is, a more stably tuned neuron would also tend to have higher information measures over more days and thus higher session-averaged information measures. We have clarified this in the corresponding methods section (‘Statistical relevance of predictors of drift’).

Our choice of stability metric as a non-time-varying value, averaged over all session pairs, is based on the assumption that the individual session-to-session difference in tuning does not suitably capture how faithfully a neuron responds to an external variable. Consider the case of a neuron that suddenly and drastically changes its tuning once over the course of a week of daily experimental sessions. This neuron’s stability would be high at every time point except one. For a population consisting of some fraction of neurons of this type and some fraction perfectly stable (same tuning on every session), the single-session stability would describe the majority of neurons as highly stable and assign only a small fraction a low stability. Our method of averaging over session pairs would instead divide the population into highly stable and moderately stable subpopulations.

In practice, while the regression results we obtained with the session-averaged stability are fairly consistent across mice and datasets (as shown in the paper), the results are less consistent with the single-session stability. Many regressions result in coefficients of 0 for a majority of variables (also in the stepwise regression analysis we newly implemented in response to the preceding comment). We have opted to not include figures showing these results, as they do not contribute any helpful clarification to the existing analyses.

However, the study you have highlighted is very interesting and relevant, and we have added a citation to it in the introduction.

In limited sampling scenarios, PID measures are biased and the bias affect the components in a different way, with the synergy being more affected (Venkatesh et al., Neurips 2023, Lorenz et al., Plos CompBio 2025); did the authors control for this/took this into account (even with some shuffling method)?

We have now applied shuffling subtraction to account for bias in the PID measures. The bias in our calculations was modest, and the bias correction has not qualitatively changed our results.

I have a remark on the notation: typically uppercase letters are used to random variables, while lowercase letters denote their realization; is there a reason why they are reversed here?

We have changed the notation to match convention. Uppercase letters now represent random variables and lowercase their realisation.

The github link is broken and I cannot find the code; maybe they forgot to make the repo public?

The github repo is now public. New code for the stepwise regression and network analysis are not yet in the repo, but we will make this available soon.

**Reviewer 2**

***1 Major comments***

The discussion contains some statements that are not backed up by the evidence presented in the Results section. This is particularly problematic because these appear to be the key conclusions that the paper attempts to draw from the results.

***1.1 Existence of redundant cliques***

> Our results demonstrate that the most stable cells in a population tend to participate in redundantly connected cliques that in turn synergistically connect to the rest of the population. Additionally, among these highly redundant subpopulations, those neurons with greater synergistic coupling to the population tend to be less stable

I do not see how this follows from what was presented above. There is nothing in the results about cliques (or other network/graph theory concepts), how they may be defined or measured, or their tendency to be synergistically connected to the rest of the population. The closest thing I can find is the plot in figure 2d, which is an example for one mouse in one recording session, and that anyway is only an illustration. There is no attempt at formalizing what it would mean for such cliques to exist and showing some quantitative evidence for that hypothesis, or that synergistic connections are indeed more common between neurons belonging to different cliques (as opposed, I assume, to between neurons within the same clique, or pairs of neurons such that one belongs to a clique but the other doesn't, or pairs of neurons that do not belong to any clique). Overall, this passage reads to me as a bit of (interesting!) speculation that should either be clearly marked as such, therefore narrowing the scope of the paper's conclusions, or developed more into an hypothesis that can actually be tested.

We have amended this statement as, ‘Our results demonstrate that the most stable cells in a population tend to participate in redundantly connected cliques that in turn synergistically connect to the rest of the population’, and we have included additional graph analysis to support this statement. The details are provided in a new methods section (‘Clique detection and graph analysis’).

In short, we detected cliques (fully connected subgraphs, illustrated in Fig S4(a)) in the redundant and synergistic graphs and compared their average stability. We then compared the average stability of the nearest neighbours of the cliques in their opposing graphs (synergistic neighbours of redundant cliques and vice versa, schematically illustrated in Fig S4(c)). We found that neurons forming redundant cliques tend to have higher stability than those forming synergistic cliques (Fig S4(b), Table S1), and that the neighbours of redundant cliques in the synergistic graph tend to have lower stability than the neighbours of synergistic cliques in the redundant graph (Fig S4(d)).

***1.2 Possible origins of redundant coupling***

> A closer look at select pairs of neurons suggests that redundant coupling may arise from either structural connections or tuning relationships among other unobserved variables.

I do not see where this closer look is taken in the Results. What selected pairs of neurons? What is the evidence presented for these pairs of neurons supporting the hypothesis that redundancy is due to structural connections or tuning relationships among other variables? and finally, these seem like very broad scenarios, so broad to be borderline uninformative: what are some alternative hypotheses? in other words, if redundancy is not due to either structural connections or tuning relationships, what else could it be due to? it would be good to provide some examples to clarify why this is an interesting hypothesis to explore rather than just a general property of redundant random variables connected in a causal network, which could either share common inputs or have direct interactions.

We have unpacked this statement in the discussion and added two supplemental figures (Figures S4 and S5) illustrating the relationships among three select pairs of neurons with high redundancy and synergy. In this data, it is likely that both structural connections and secondary tuning relationships are responsible for the redundancy and synergy we observe, and we wished to allow for both of these interpretations. With the added supplementary figures, we provide some evidence for each.

***1.3 Unclear support for the idea of "generally salient task features"***

> [..] we conjecture that redundancy reveals how drift is constrained across the population in a way that limits interference with relationships among task variables that are more generally relevant across many tasks. […] this work provides compelling evidence that stable subsets of the population encode for generally salient task features, evident in the stimulus-conditioned relationships among the neuronal responses.

I have multiple issues with this passage. First, I think it is not clear what exactly is meant by "generally salient task features" here, which is not a good thing given that the passage claims that the paper offers compelling evidence for something involving those features. What is an example of a generally salient task feature? Second, my best guess for its meaning from the broader context in the paragraph is that "generally salient features" is just another way of saying "task variables that are generally relevant across many tasks", which is a much clearer concept. Assuming that is the case, I do not understand how the present work could possibly provide any evidence (let alone "compelling" evidence) for this, given that, of the two experiments analyzed, one had only one task and the other had no task at all. Either this claim is vastly overstated, or there is some key logical passage not well explained in the results, that from the distribution of the regression coefficients in figures 2b,c (essentially the only relevant pieces of quantitative evidence) allows to jump to what seems like such a far-fetched conclusion.

We intended for this to be a conjectural interpretation of our results, rather than a conclusion definitively supported by this work. We have softened the language around this claim and provided more detail to unpack our conjecture.

*“On the basis of these observations, we conjecture that redundancy may reveal how drift is constrained across the population in a way that limits interference with relationships among task variables that are more generally relevant across many tasks. The stimulus- or behaviour-conditioned co-activation of highly redundant neuron pairs suggests that either they are structurally connected or they are similarly tuned to a second external variable. In the former case, their connection may serve a reinforcing effect, essentially making it harder for their tuning to be disturbed by outside fluctuations to only one neuron of the pair. In the latter case, neuron pairs being similarly responsive to not only the target task variable but also a second unobserved variable indicates this combination of task variables recruits more neurons to represent it, which is suggestive of high salience.”*

***2 Minor***

***2.1 Clarity of the central claim***

There seems to be some misalignment in the emphasis placed on different claims between the abstract and the discussion. As mentioned above, the discussion places emphasis on the differences between how redundancy and synergy are distributed in the neural population, while the abstract reads as follows:

> We found that a neuron’s tuning stability is positively correlated with the strength of its average pairwise redundancy with the population, and that these high-redundancy neurons also tend to show high average pairwise synergy

Picking one central claim, sticking by it and ensuring it is well supported by the data would make the paper much more impactful in my view.

We have removed the secondary claim from the abstract to emphasize our main claim of the relationship between stability and redundancy.

***2.2 Oversimplified explanation/example of synergy***

The following passage seems oversimplified:

> For example, a pair of neurons with shared tuning to a second external variable v would show high redundancy, as would a pair of neurons that mutually excite one another. In contrast, a pair of neurons with dissimilar tuning to v and a mutually inhibiting pair would show high synergy.

While the statements about redundancy seem correct, that on synergy seems potentially misleading. I agree with the sentiment that it is easy to construct toy examples of mutually-inhibiting model neurons that code synergistically for some external variable, but this does not immediately imply that such pairs always have high synergy. Consider the following example, where v is taken as an external variable and x, y are the activity states of two neurons:

━━━━━━━━━━

v x y p(x,y|v)

────────────────────

0 0 0 ¾

0 0 1 ¼

0 1 0 0

0 1 1 0

────────────────────

1 0 0 ¾

1 0 1 0

1 1 0 ¼

1 1 1 0

━━━━━━━━━━

In this scenario, the neurons are mutually inhibiting in the sense that the (1,1) state never occurs, and they have "dissimilar tuning" in the sense that x is tuned for v=1 and y is tuned for v=0. But it doesn't seem like there should be any synergy, because for both values of the variable v there is one of the two neurons such that observing that neuron will determine the value of v with certainty (in other words, there is no information in the system that is accessible only by simultaneous observation of the two neurons)

If this counterexample is correct, I don't think that this passage is particularly helpful for the general reader who may not be familiar with PID. I suggest simply modifying the passage to state that neurons with mutual inhibition may exhibit synergy, perhaps adding a couple of toy examples in a supplemental figure to show when this may or may not be true.

There is a crucial point to make clear in this example: the redundant and synergistic information are taken with respect to a given external variable and are thus different when considering a different external variable in the task—i.e., synergy wrt u ≠ synergy wrt v. We have added the toy example below to Appendix A as support for our statement in the main text.

In the toy example in the manuscript, the synergy is with respect to the variable u, to which the pair exhibit similar tuning, and one potential source of this synergy could be the neurons’ dissimilar tuning to a second variable v. To relate this toy example to one of the datasets we analysed (Driscoll et al. 2017), let us say v={0,1} indicates that a trial is a left- or right-turn trial, respectively, and u is the position, with two position bins for the sake of simplicity.

We can consider the probabilities from the table above, where neuron x is active in only right-turn trials, and neuron y in only left-turn trials. We can assign them the same position tuning by making them active only in position bin u=0 as follows:

━━━━━━━━━━

u x y p(x,y|u)

────────────────────

0 0 0 ½

0 0 1 ¼

0 1 0 ¼

0 1 1 0

────────────────────

1 0 0 1

1 0 1 0

1 1 0 0

1 1 1 0

━━━━━━━━━━

This table preserves the active probabilities p(x) and p(y) from the table for v, and we assume u={0,1} and v={0,1} occur independently with equal probabilities. We can also create an expanded probability table based on these two variables.

━━━━━━━━━━━━━━

v = 0	v = 1

u x y p(x,y|u,v)	u x y p(x,y|u,v)

────────────────────────────

0 0 0 ½	0 0 0 ½

0 0 1 ¼	0 0 1 0

0 1 0 0	0 1 0 ¼

0 1 1 0	0 1 1 0

────────────────────────────

1 0 0 1	0 0 0 1

1 0 1 0	1 0 1 0

1 1 0 0	1 1 0 0

1 1 1 0	1 1 1 0

━━━━━━━━━━━━━━

First, an intuition-based explanation. The toy example here resembles a discretised version of the activity shown in Fig. 1(h) and (i): when the animal is in position u=1 (yellow), the neurons are inactive (x=0, y=0), and in position u=0 (purple), one or the other is active (x=1, y=0 or x=0, y=1). This has the effect of better separating the regions of xy space where u=0 and u=1 than if the activity of x and y were uncorrelated, as illustrated in Fig. 1(i).

For a more mathematically rigorous explanation, we can calculate the synergistic from Eq. (9) in the Methods, using the formulation by Bertschinger et al.:

$$I_{syn}\left( u:x,y \right)=I\left( u:x,y \right)-\min_{Q\in\Delta_{P}} I_{Q}\left( u:x,y \right).$$

We can calculate the joint mutual information from the probability table for u as $I\left( u:x,y \right)=\frac{3}{2}-\frac{3}{4}\log_{2} 3=0.31 \mathrm{bit}$. The second term is the minimal joint mutual information among the set of probability distributions $\Delta_{P}$ that maintains the tuning curve relationships between each neuron and u (i.e., the marginal distributions p(u,x) and p(u,y) are the same). This distribution would be the case where the two neurons are always active together in the position bin to which they are tuned:

━━━━━━━━━━

u x y p_Q_(x,y|u)

────────────────────

0 0 0 3/8

0 0 1 0

0 1 0 0

0 1 1 1/8

────────────────────

1 0 0 1

1 0 1 0

1 1 0 0

1 1 1 0

━━━━━━━━━━

The joint mutual information for this probability distribution is $I_{Q}\left( u:x,y \right)=2+\frac{3}{8}\log_{2} 3-\frac{7}{8}\log_{2} 7=0.14 \mathrm{bit}$. The synergistic information is then the difference between these two, which gives us $I_{syn}=0.17 \mathrm{bit}$.

Note that the same results come from assuming the two neurons mutually inhibit one another, as the probability table for u would be the same.

***2.3 Statistical impact of multiple-counting of neurons recorded in different days***

If I understand correctly, for the analyses in the paper (for example in the regression) a neuron recorded over multiple days is counted as multiple, statistically independent datapoints. If this is the case, this seems like a very strong assumption at best; it should be at least highlighted in the methods and its potential implications on the conclusions drawn from the regression analyses discussed.

We have added clarifying details about the regression method to the corresponding methods section (‘Statistical relevance of predictors of drift’). We performed separate regressions on each session, so that for each regression, a neuron was represented only once. The predictors were single-session information metrics, and the response variable was the tuning stability averaged over session pairs. The data points in Figure 2(b) and (c) each correspond to the coefficients a single regression (i.e. a single session from one mouse).

***2.4 Details on statistical inference***

The paper mentions that the regression analysis is performed using elastic net with α=0.5, but several key details are missing.

• what is the reason for chosing this particular value of α?

• how is λ selected? the methods say "We selected the λ parameter corresponding to the model with the lowest mean squared error to obtain the final coefficient values", but this is ambiguous. This process probably involved cross-validating the MSE on some held-out data (which is the appropriate way of doing this), but if so it should be stated, and the details of the cross-validation procedure should be given.

• the elastic net paper by Zou and Hastie should be cited.

Moreover, Figure 1 caption mentions correctly that the three predictors considered all have strong pairwise correlations with each other. Unfortunately the reader is left to read this in a fairly contrived way from Figure 1a, while it could be made evident at a glance in a simple pairplot. Please provide a pairplot of the predictors (for instance as a supplementary figure).

We have added the missing methodological details and citation to the corresponding methods section (‘Statistical relevance of predictors of drift’), including the MATLAB function that was used for the elastic net analysis. We performed the regression with α values of 0, 0.5, and 1 and found that the regression coefficients showed little dependence on the α value selection. For the selection of λ, we used the minimum 10-fold cross-validated MSE. We have also added pair plots of the regressors as Supplementary Figure S1.

***2.5 Impact of binning:***

Around line 301 the text says: "Limitations of the data indicated a coarse bin size, which we verified was sufficient to support our conclusions."

It is not immediately clear from this passage how this was verified, and how it would have been possible to detect if it was otherwise. Please expand.

We confirmed that our analysis produced similar results at different bin resolutions. As detailed later in the same section, we binned the Driscoll data into 5, 10, and 20 bins, and the latter two bin resolutions yielded similar results.

***2.6 Typos / small edits***

• Figure 1 caption: "We binned the position is binned into…"

• lines 171-172: "Fig 2(b) and (c) shows the regression coefficients for the three regressors for PPC tuning to position in the maze and heading of the animal and for V1 tuning to the drifting gratings and naturalistic movies, respectively." This passage should probably read "…for PPC tuning *stability*…", etc.

• Please cite the Dryad repo also for Marks and Goard.

We have made these minor edits.

**Reviewer 3**

***Major comments***

***1. Working Hypothesis***

I have concerns about the working hypotheses proposed in the introduction. As I understand it, the authors suggest that neurons conveying redundant information about core task or stimulus features are more constrained and stable over time, and thus less susceptible to representational drift. However, I would argue that neurons conveying highly redundant information could still exhibit strong representational drift, provided that their activity changes in a similar way—for instance, due to modulation over time of shared inputs. Conversely, one could argue that neurons conveying strong synergistic information about task variables should show greater stability over time. Given their heterogeneous coding, even slight representational shifts could cause the synergistic information to diminish.

In other words, the current working hypothesis does not appear to be strongly supported by empirical evidence or theoretical rationale. I suggest revising the final paragraph of the introduction to acknowledge and account for these alternative hypotheses. The question addressed in the paper is important, but I find the working hypothesis less convincing in its current form.

We have revisited the final paragraph of the introduction. In the final paragraph, we clarify that the main finding of our study is this positive correlation between redundancy index and tuning stability, and we clarify that the further hypotheses we present in the final sentences of the introduction are regarding potential mechanisms that we believe may underlie this relationship.

***2. Statistical Analysis of Information Measures***

The manuscript focuses on highly redundant and synergistic neuron pairs. However, no statistical analysis was performed to determine the significance of the main effects or to validate the identification of these neuron pairs. A common approach to assess significance in PID measures is to use surrogate data by shuffling the target variable (e.g., task-related variables). Additionally, any analysis should account for multiple comparisons, for example by applying FDR or Bonferroni corrections. The authors should consider performing such analyses or using quantitative methods to support their claims about highly redundant/synergistic neuron pairs.

We have now applied shuffling subtraction to account for bias in the PID measures. The bias in our calculations was modest, and the bias correction has not qualitatively changed our results.

To our understanding, the problem of multiple comparisons is not applicable to the regressions performed here. Multiple comparisons involve comparisons between a response variable and multiple independent predictor variables, whereas here we are dealing with multicollinear rather than independent predictors. Our choice of regularised regression accounts for the problem of multicollinearity.

***3. Figure 1***

Panel (a) contains a large amount of information, and the corresponding description in the results section is neither linear nor clear. The text reads more like a figure legend than a results narrative. I recommend rewriting this section to clearly describe the main results and streamline the narrative. Also, consider removing non-essential information (e.g., panel c). The definition of highly redundant pairs appears to overlap with standard linear correlation (see Fig. 1d). However, this might not always be the case. For example, panels (e) and (i) show correlation and anti-correlation, both of which could correspond to redundant information.

Additionally, panel (b) introduces the Redundancy Index before it is defined in the main text. Overall, I suggest revisiting Figure 1 entirely—selecting the most relevant results and providing a clearer, more structured explanation in the results section.

We have chosen to keep Fig. 1 largely unchanged, though we did remove the colour scale in the stability histograms in Fig. 1(b). We believe the experimental schematic in Fig. 1(a) and corresponding explanation, and the classic PID schematic in Fig. 1(c) are helpful for introducing unfamiliar readers to these important components of our work. We have aimed to keep experimental details as concise as possible while conveying sufficient information for a reader unfamiliar with these experiments to follow along with the important points of our analysis. The example pairs of neurons shown in Fig. 1(d) and (h) are not meant to be definitions of redundancy and synergy but illustrative of the types of correlations we observe in neuron pairs in the datasets with high redundancy and synergy, and we have clarified this in the text. We have added a supplementary figure (Fig. S5) that shows the activity of these pairs on other sessions, along with their tuning curves and redundant and synergistic information over all sessions.

***4. Statistical Relevance of Predictors of Drift***

The description of the regularized regression procedure is insufficient. The manuscript should provide a more thorough and accessible explanation of the method and include references. For example, it should be clarified that the elastic net minimizes a specific loss function, combining L1 and L2 penalties. It is particularly useful for constructing linear models when predictors are numerous and potentially correlated.

We have added additional methodological details and references to the corresponding methods section (‘Statistical relevance of predictors of drift’) and clarified the motivation for choosing elastic net in the main text.

***5. Synergistic Information and Representational Drift***

The negative correlation between synergistic information and representational drift is intriguing and could represent an important result. However, the manuscript emphasizes redundancy encoding results, and this potentially significant finding receives little attention. I suggest the authors highlight and elaborate on this finding more thoroughly.

We have added a paragraph on the implications of synergy and drift to the discussion section.

*“In contrast to the case of redundancy, high-synergy pairs arising from structural connections would likely involve mutually inhibition. It is possible that such pairings would produce stimulus-specific competition, driving more changes in tuning than would occur without such interactions. It is widely considered that synergy is a advantageous approach to information encoding in the brain, as it takes advantage of the full range of combinations of activations in the system; however, it is also fragile and susceptible to disruption by a small number of failures in the network (Luppi et al. 2024). This suggests that while it would be valuable to exploit synergistic interactions in the neural code, it may also be a good strategy for the brain to allow some flexibility in reading out information from neurons with many such interactions.”*

***6. Conclusion***

The conclusion is currently very brief and does not reflect the full scope of the main findings. A more comprehensive conclusion summarizing key points—including those related to synergistic coding and the statistical predictors of drift—would strengthen the manuscript.

We have added a brief conclusion section where we summarise the main results.

*“In this study, we evaluated the relationship among redundancy, synergy, and tuning curve stability in two longitudinal neural datasets. We found that neurons with higher redundant coupling to the population tend to exhibit higher tuning stability. Neurons tend to organise into highly redundant and highly stable cliques that in turn synergistically connect to the remainder of the population. We also found that synergy tends to be negatively correlated with tuning stability. We theorise that neurons with high redundant coupling to the population may be tuned to highly salient aspects of the task, motivating their more stable relationship to task variables, whereas neurons with high synergistic coupling may be driven to drift more as a result of competition. Regardless of the mechanism underlying our results, our finding can benefit work where the relationships between neural activity and task variables must be tracked over long timespans, such as brain--machine interface research.”*

***Minor issues***

Important references on Partial Information Decomposition (PID) are missing.

We have added additional references to PID literature, including Williams and Beer (2010) and Wibral et al. (2017).

The code availability section lacks the link: <https://github.com/kris-heiney/PID-Drift/>

The github repo is now public. New code for the stepwise regression and network analysis are not yet in the repo, but we will make this available soon.
