# Supplementary material for: Information theoretic measures of neural and behavioural coupling predict representational drift
Source: PLoS Comput Biol. 2026 Feb 17;22(2):e1013130. doi: 10.1371/journal.pcbi.1013130 (PMC12952586; doi:10.1371/journal.pcbi.1013130)
Supplement: Attachment — Submitted filename: Response_to_reviewers_auresp_2.docx [file pcbi.1013130.s012.docx]

**Response to reviewers**

**Journal**: PLOS Computational Biology

**Manuscript**: Information theoretic measures of neural and behavioural coupling predict representational drift

**Authors**: Kristine Heiney, Mónika Józsa, Michael E. Rule, Henning Sprekeler, Stefano Nichele, Timothy O’Leary

Thank you for the close engagement with our paper. We have revised the manuscript extensively and respond to comments below.

**Reviewer 1**

Lines 106–109 of the revised manuscript, in which examples of synergy and redundancy are proposed, might be confusing. A pair of neurons with dissimilar tuning might generate synergy, but the example in Fig 1 involves neurons with similar tuning (both towards 0). I think the easiest way to explain this to a naive reader is through noise correlations at a fixed stimulus (as done in Fig 1 indeed).

In the examples for both redundancy and synergy, the pair of neurons indeed have similar tuning to the target external variable U; the difference is in the second unobserved variable V, which is what causes the noise correlations you mention. This example corresponds to the illustration in Fig 1 and the toy example in Appendix A, written in response to Reviewer 2 in the first round of revisions.

To help clarify the intended toy examples, we have added a diagram showing the cases we indicate in the text as Figure 1(c). This new diagram replaces the partial information decomposition diagram, following the recommendation from Reviewer 3 in the first round of revisions.

I would consider a third figure combining the right part of Fig 2 and Fig S4, to give more emphasis to the network result, which is quite nice in my opinion.

We appreciate the interest in the network results and have made a third figure as you suggested.

**Reviewer #2**

1. Regression analyses

A. what is the motivation for performing a stepwise regression? why not simply add the interaction coefficients to the elastic net models, as suggested by R1?

We have changed the regression method to include interaction terms in the elastic net regression analysis.

B. The text says "In the PPC dataset with U the position, when the redundancy index × synergy index term was present, it tended to be negative". I do not see how one can conclude that from Figure S2 (for instance looking at the pooled data for all mice). Figure S2 shows that the red:syn interaction is generally zero, and that the MI:red interaction is generally negative. Looking at the other plots in figure S3 it is hard to convince oneself that there is anything that makes the distribution of the red:syn parameter estimates of PPC/position more interesting than the other interaction terms. Can you clarify this point?

We have removed this claim and focused on interpretations more definitively supported by the regression analysis.

C. Even disregarding the previous point, I don’t fully agree with the interpretation of the results of the regression with interaction terms. Consider the sentence “among neurons with a given redundancy index, a neuron with a higher synergy index would tend to exhibit lower tuning stability”. The correct interpretation of the interaction term is that, if the interaction is negative, it means that if you partition the neurons in low-redundancy and high-redundancy then the high-redundancy ones will have a synergy-stability relationship with a smaller slope than the ones with low redundancy. But this is not the same thing as saying that the slope must be negative (“a neuron with a higher synergy index would tend to exhibit lower tuning stability”). Let me expand. Consider, in general, a linear model with two predictors, X1 and X2, and target variable Y. Then the model with an interaction is Y=β₁X₁+β₂X₂+β₃X₁X₂, so that when one fixes X₂ (the redundancy in our case) one is left with Y=(β₁+β₃X₂)X₁+β₂X₂. This is a linear relationship with slope (β₁+β₃X₂) and intercept β₂X₂. So whether the slope is positive or negative (that is, whether neurons with higher synergy will have lower stability) depends on both β₁ and β₃. In the case at hand, β₁ is generally negative, but it would be better to specify in the text that the effect holds because of the interplay of the two terms, especially as this is important to keep in mind when trying to interpret the other plots in figure S3.

Thank you for this thorough explanation. We have decided to limit our interpretation of the regression analysis to avoid speculating about interactions between the predictors, as the results do not provide strong evidence to support any such claims.

D. Something I missed in the first round is related to the following sentence in the methods: "If any predictor did not have a significant correlation with the stability in a single-variable model (i.e., p > 0.05), its regression coefficient was set to 0." Can you expand on this? My understanding is that (a) Elastic Net does not allow for a closed-form sampling distribution (even an asymptotic one) of the parameter estimates under the null hypothesis, so I don't understand how these p-values were computed, unless there was something more complex going on that was not described in the Methods (such a permutation test of some sort). (b) it is highly unusual to manually threshold parameter estimates in this way, at least in elastic net, because the L1 component of the regularizer should automatically take care of setting irrelevant parameters exactly to zero. So I struggle to see the statistical rationale behind this operation.

We have removed this step of setting coefficients to zero in the regression process. All coefficient plots reflect this revised method, and the results are not qualitatively affected by this change.

2. Clique analysis

A. If I understand correctly, the p-values reported in Table S1 come from computing a Wilcoxon signed-rank test to the data that is displayed as dots in Figure S4 b,d. Each datapoint is two-dimensional and it is calculated as (for instance) the stability of the nodes in redundant and synergistic cliques that can be found in a session. For each session there are many datapoints because each of them corresponds to a different choice of the binarization threshold for the graph, used in finding the cliques. One problem I see with this procedure is that the datapoints thus determined are clearly not statistically independent, and thus do not satisfy the assumptions of the Wilcoxon test. To show why this is the case, imagine what happens if the increments of the threshold "grid" were to become very small: at some point one would start getting datapoints that are identical copies of each other, because in general the binarization-and-clique-finding process will have some degree of robustness to the choice of binarization process (meaning that the cliques in the binarized network, and essentially the network itself, will be the same for threshold t and t+ε, with ε small). Whether there are choices of the increments for this parameter that guarantee that networks obtained with different thresholds can be considered as independent random samples from some underlying distribution is not obvious to me, a priori.

We agree that the data points derived from multiple thresholds are not statistically independent. Rather than apply a parametric statistical test, we opted to use bootstrapping, as described in detail in the revised Methods section. The conclusions we draw from these new statistical results are qualitatively similar to those in the previous version of the manuscript.

For each mouse, we produced 1,000 bootstrapped replicates of the two graph quantities we considered in the analysis: the mean clique stability and the mean nearest neighbor stability. Figure S4(a,c) shows histograms of the means of these replicates, pooled over mice, and Figure S4(b,d) shows the 95% confidence intervals per mouse.

For the mean clique stability, the redundant cliques generally exhibited greater stability than their size-matched synergistic counterparts, with a difference of 0 lying outside of the 95% bootstrap confidence interval in all but three cases ((i) U = position, mouse 3, sessions 13-22, right turns; (ii) U = heading, mouse 3, sessions 13-22, right turns; (iii) U = movies, mouse 10). For the mean nearest neighbor stability, the synergistic neighbors of the redundant cliques exhibited lower stability than the redundant neighbors of the synergistic cliques, with a difference of 0 lying outside the 95% bootstrap confidence intervals in all cases.

These results support our statements that redundant cliques tend to be highly stable, and that these stable cliques connect synergistically to lower-stability neurons in the population.

B. Tangentially, table S4 contains 32 statistical tests and I couldn’t find a mention of the multiple-test correction that was used.

We have addressed this with our alternative approach to assessing statistical significance via bootstrapping.

C. I get the reasoning behind studying the connectivity between nodes in a clique and the nearest neighbors of the clique, but I don't understand why this is studied always in the opposing graph (synergistic graph for the redundant cliques and redundant graph for the synergistic cliques). This should be explained in the text.

We have moved the network figure into the main text, as recommended by Reviewer 1. In the new expanded explanation of the figure in the main text, we give more details of the reasoning behind our choices. We use the graph analysis to support two claims: (1) that the most stable neurons form redundant cliques, and (2) that these cliques are connected to the rest of the population synergistically. To this end, we consider (1) the mean stability of redundant cliques, and (2) the mean stability of their nearest synergistic neighbors. To have a point of comparison, we use (1) the mean stability of synergistic cliques and (2) the mean stability of their nearest redundant neighbors as a sort of null model.

D. Regarding the summary sentence for the clique results paragraph: "This indicates that highly stable neurons tend to redundantly couple to each other and synergistically couple to other lower-stability neurons in the population". I think the results, at face value (modulo my concerns above), show that neurons in redundant cliques tend to have higher stability. I don't think this is the same as saying that stable neurons tend to redundantly couple with each other. Is it possible, for instance, that highly stable neurons also tend to participate in very few cliques? in that scenario, it would not be correct to say that highly stable neurons tend to connect to each other in such way, because they primarily would simply tend to connect less (or connect in ways that do not form cliques). Perhaps the data in the other figures already excludes this possibility, but if it is the case it would be good to say that explicitly. More generally, the sentence I am commenting on here tries to draw a conclusion on how stability influences redundancy and synergy, while the analyses go in the opposite logical direction, so it seems that some additional care is required.

We have rewritten this section to more accurately reflect the implications of our graph results.
